# Adaptive Mobile Health Intervention for Adolescents with Asthma: Iterative User-Centered Development

**DOI:** 10.2196/18400

**Published:** 2020-05-06

**Authors:** David A Fedele, Christopher C Cushing, Natalie Koskela-Staples, Susana R Patton, Elizabeth L McQuaid, Joshua M Smyth, Sreekala Prabhakaran, Selina Gierer, Arthur M Nezu

**Affiliations:** 1 Department of Clinical & Health Psychology University of Florida Gainesville, FL United States; 2 Clinical Child Psychology Program & Schiefelbusch Institute for Life Span Studies University of Kansas Lawrence, KS United States; 3 Nemours Center for Healthcare Delivery Science Nemours Children's Health System Jacksonville, FL United States; 4 Department of Psychiatry and Human Behavior Brown University Providence, RI United States; 5 Department of Biobehavioral Health Pennsylvania State University University Park, PA United States; 6 Department of Pediatrics University of Florida Gainesville, FL United States; 7 Department of Pediatrics University of Kansas Medical Center Kansas City, MO United States; 8 Department of Psychology Drexel University Philadelphia, PA United States

**Keywords:** asthma, mobile health, adherence, adolescence, self-regulation, problem-solving, adolescent, youth

## Abstract

**Background:**

Adolescents diagnosed with persistent asthma commonly take less than 50% of their prescribed inhaled corticosteroids (ICS), placing them at risk for asthma-related morbidity. Adolescents’ difficulties with adherence occur in the context of normative developmental changes (eg, increased responsibility for disease management) and rely upon still developing self-regulation and problem-solving skills that are integral for asthma self-management. We developed an adaptive mobile health system, Responsive Asthma Care for Teens (ReACT), that facilitates self-regulation and problem-solving skills during times when adolescents’ objectively measured ICS adherence data indicate suboptimal rates of medication use.

**Objective:**

The current paper describes our user-centered and evidence-based design process in developing ReACT. We explain how we leveraged a combination of individual interviews, national crowdsourced feedback, and an advisory board comprised of target users to develop the intervention content.

**Methods:**

We developed ReACT over a 15-month period using one-on-one interviews with target ReACT users (n=20), national crowdsourcing (n=257), and an advisory board (n=4) to refine content. Participants included 13-17–year-olds with asthma and their caregivers. A total of 280 adolescents and their caregivers participated in at least one stage of ReACT development.

**Results:**

Consistent with self-regulation theory, adolescents identified a variety of salient intrapersonal (eg, forgetfulness, mood) and external (eg, changes in routine) barriers to ICS use during individual interviews. Adolescents viewed the majority of ReACT intervention content (514/555 messages, 93%) favorably during the crowdsourcing phase, and the advisory board helped to refine the content that did not receive favorable feedback during crowdsourcing. Additionally, the advisory board provided suggestions for improving additional components of ReACT (eg, videos, message flow).

**Conclusions:**

ReACT involved stakeholders via qualitative approaches and crowdsourcing throughout the creation and refinement of intervention content. The feedback we received from participants largely supported ReACT’s emphasis on providing adaptive and personalized intervention content to facilitate self-regulation and problem-solving skills, and the research team successfully completed the recommended refinements to the intervention content during the iterative development process.

## Introduction

### Background

Over 8% of youth have an asthma diagnosis, making it the most prevalent pediatric chronic illness [1]. Asthma is a leading cause of emergency department visits, missed school days, and healthcare expenditures, making it a significant public health concern [2,3]. Youth can mitigate asthma-related morbidity via consistent engagement in a complex set of daily disease self-management behaviors (eg, monitoring symptoms, avoiding triggers). According to national guidelines, adherence to inhaled corticosteroids (ICS), medications designed to control asthma and reduce the likelihood of exacerbations, is critical to asthma self-management for youth with persistent asthma [4]. High levels of adherence to ICS (ie, taking >80% of prescribed doses) are associated with both consistent asthma control in youth [5] and reduction in severe asthma exacerbations [6].

Adolescence is a unique developmental period when suboptimal adherence to ICS is common [7-9]. As adolescents’ responsibility for disease management (eg, taking ICS) increases, there is less direct caregiver contact and an increased desire for autonomy [8]. Concurrently, adolescents’ executive function abilities that undergird the self-regulation and problem-solving skills integral for successful asthma self-management are not fully developed [10-15]. Together, these factors put adolescents at high risk for asthma-related morbidity and reduced quality of life [16-19]. There are evidence-based face-to-face programs available addressing adolescent adherence; however, there are often numerous barriers to implementation including infrequent encounters at the point of care and logistical obstacles (eg, transportation, absence of trained interventionists) [20]. Thus, there continues to be a large number of youth who do not take their medication as prescribed [21], leading many researchers to pursue novel intervention frameworks for improving adherence among adolescents.

Mobile health (mHealth) interventions have recently received considerable attention for improving adherence to ICS among youth [20,22]. Smartphones are a readily available intervention medium for youth with asthma given their ubiquitous nature across socioeconomic strata and habitual daily use by adolescents [23,24]. Furthermore, recent advances in ambulatory disease management technology (eg, medication sensors) have led to opportunities in mHealth for continuous passive monitoring to better identify and contextualize states of vulnerability for poor or declining disease self-management. These technological advances have led to burgeoning interest in the development of adaptive mHealth interventions where youth could receive in-the-moment support during periods when a person with a disease may be most in need [25]. To our knowledge, no adaptive mHealth systems exist that objectively monitor youth adherence to ICS and use that data to deliver a tailored, timely, and theory-based intervention.

Our interdisciplinary team of behavioral health experts, pediatric pulmonologists, and technologists recently developed an adaptive mHealth adherence promotion intervention, Responsive Asthma Care for Teens (ReACT), that is grounded in self-regulation theory (SRT) [23]. ReACT is an integrated mHealth system that passively monitors adolescents’ adherence to ICS using a novel Bluetooth-enabled sensor, developed in collaboration with the University of Kansas Instrumentation Design Laboratory, that attaches to ICS canisters. ReACT activates when an adolescent’s ICS adherence data indicate a clinically-derived need (ie, <80% [5]) and delivers tailored intervention content via the Way to Health text messaging platform. Once active, ReACT uses 2 components, a goal-setting algorithm and tailored problem-solving modules, to support adolescents’ self-reaction to suboptimal adherence and improve self-efficacy. ReACT’s goal-setting algorithm aids adolescents in self-monitoring, feedback, and goal setting via gain-framed [24] messages. ReACT uses algorithms that assess an adolescent’s recent patterns of adherence (eg, trajectory, patterns of missing doses) in order to deliver meaningful goal intention–formatted message content. For example, at times when adherence is suboptimal, ReACT delivers a brief motivation assessment. If upon completing this assessment, the adolescent endorses motivation to take at least some of his/her medication, then ReACT will ask the adolescent to report any intrapersonal or external barriers to ICS adherence (eg, stress) so that ReACT can deliver a tailored problem-solving module while also continuing to monitor the adolescent’s ICS use and provide adherence feedback and goal-setting content. Alternatively, if an adolescent indicates that he or she is not currently interesting in taking their ICS, then ReACT provides educational content focused on the importance of medication adherence and delays restarting the goal-setting algorithm for 3 days, giving the adolescent time to process ReACT’s educational message.

### Current Study

This study illustrates the iterative user-centered design process we used in developing ReACT, which is consistent with best practices for the development of mHealth pediatric intervention content [26]. Specifically, we describe how we leveraged a combination of individual interviews, national crowdsourced feedback, and an advisory board comprised of target users to develop the core ReACT intervention content and supporting technical and device-related infrastructure. All study procedures were approved by the Institutional Review Boards at the University of Florida and University of Kansas.

## Methods

### Study Stages

**Figure 1:**
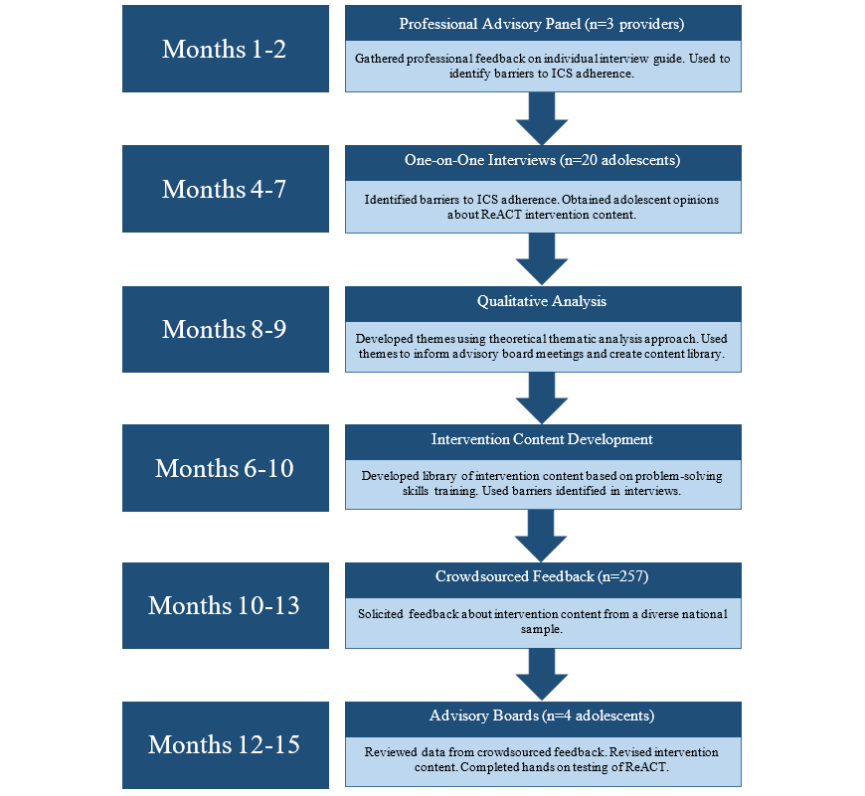
Study overview and timeline. ICS: inhaled corticosteroids; ReACT: Responsive Asthma Care for Teens.

Our intervention development process consisted of 6 stages, with refinements occurring between iterations. Figure 1 provides an overview of the study procedures and timeline.

### Participants

Participants included 13-17–year-olds with asthma and their caregivers. Individual interviews were completed by 20 adolescent-caregiver dyads (10 from the University of Florida and 10 from the University of Kansas Medical Center), 257 adolescents provided crowdsourced feedback via a national online panel, and 4 adolescent-caregiver dyads participated in advisory board meetings. One dyad participated in both individual interviews and advisory board meetings, yielding a total of 280 adolescents and 23 caregivers. Information regarding recruitment is provided below. For individual interviews and advisory board meetings, dyads were eligible to participate if the adolescent had a physician-verified diagnosis of asthma with persistent symptoms requiring ICS use for at least 6 months, the adolescent had a daily ICS or ICS/long-acting beta agonist prescription for at least 6 months, and the adolescent and caregiver were fluent in English. We excluded dyads from individual interviews and advisory boards if the adolescent had a comorbid chronic health condition that may affect lung function (eg, cystic fibrosis) or if the adolescent had a significant cognitive impairment or developmental delay that could interfere with study completion. For crowdsourced feedback, adolescents were eligible to participate if their caregiver provided affirmative answers to the following questions: “Have you ever been told by a doctor, nurse, or other health professional that your child has asthma?” and “Does your child who is 13-17 still have asthma?” Epidemiological trials (eg, National Health Interview Survey) commonly use these questions to screen for persistent asthma [27].

### Procedure

We recruited a convenience sample of individual interview and advisory board meeting participants via clinics and flyers at both sites. At the University of Florida, we also recruited participants via a database of patients that consented to be contacted for research. We used Qualtrics, a leading online panel and survey technology provider, for crowdsourcing to ensure a nationally representative sample of adolescents with asthma in regards to race/ethnicity. Participants completed a screener that ensured that children were between the ages of 13 years and 17 years, had received a diagnosis of asthma, were physically present to participate in the survey, and still had asthma. For participants recruited via Qualtrics, we set the following quotas in order to solicit feedback from youth with asthma from a diverse range of ethnic backgrounds: 30% Black/Non-Hispanic, 24% more than one race, 16% White/Non-Hispanic, 14% Asian, 4% American Indian/Alaska Native, 4% Native Hawaiian or other Pacific Islander, 4% White/Hispanic, and 4% Black/Hispanic. We combined all quotas for non-White participants into one group to allow for faster data collection after 2 months of recruitment. Please see our previous work for more detail about the recruitment procedures [23].

#### Design Phase I

Design Phase I consisted of individual interviews with 20 adolescents diagnosed with persistent asthma. The study team, in conjunction with a professional advisory panel comprised of a pediatric pulmonologist, clinical pharmacist, and advanced practice registered nurse, created a semistructured interview guide. The guide assessed salient intrapersonal and external adherence barriers prior to the start of interviews and the types of intervention content adolescents would prefer in an mHealth intervention when encountering these barriers. The guide was informed by SRT [28], our pilot data [29], and the existing pediatric asthma literature on adherence to ICS [30,31]. We used a graduate student (NS) at the University of Florida and a research coordinator at the University of Kansas to conduct the interviews. The principal investigators (DF and CC) trained research staff in interview administration and conducted at least two mock interviews prior to conducting participant interviews. Interviews were 30-60 minutes and audiorecorded. Adolescents and their caregivers completed asthma-related questionnaires [23] in order to describe the sample and gather data on asthma-related and adherence-related constructs. Adolescent-caregiver dyads received US $60 as compensation for their participation.

A transcription service completed verbatim transcriptions of e-recordings of individual interviews. We entered interview files into NVivo (QSR International, Doncaster, Australia), a qualitative data management system. Two research assistants coded and aggregated interviews using a theoretical thematic analysis approach for developing themes [32-34]. This approach used a priori thematic categories guided by SRT, although we allowed for de novo themes to emerge from interviews. We resolved differences via discussion between research assistants and one of the principal investigators (DF). Subsequently, we used the information gathered from individual interviews, preliminary data, and the extant asthma literature to develop intervention content for ReACT.

#### Design Phase II

Design Phase II consisted of gathering feedback on ReACT intervention content via national crowdsourcing via Qualtrics and an advisory board of target ReACT users. The 7 different domains of content and branching logic included in ReACT resulted in approximately 80 pieces of content to rate per domain. Thus, we elected to allow each participant to view content from only 1 domain to reduce participant burden. An average of 33 participants (range 28-35 participants) viewed content for each domain and rated its appropriateness using a dichotomous response choice: “yes” (I like the message as it is) or “no” (change it to make it better). When crowdsourcing participants answered “no,” they had the option to reword the message to make it better. Consistent with previous research [35], content receiving ≥60% “no” votes was discarded, and content receiving ≤39% “no” votes was accepted as final content. Content receiving 40%-59% “no” votes was subject to revision. Finally, to complete this stage of content review and refinement, an advisory board comprised of 4 adolescents diagnosed with persistent asthma from the University of Florida convened 3 times over the span of 4 months to refine the intervention content based on feedback from the crowdsourcing. Advisory board members and our previous individual interview participants completed the same asthma-related questionnaires. During advisory board meetings, we audiotaped their comments and transcribed the tapes to inform ReACT design decisions. Adolescent-caregiver dyads received US $50 for each advisory board (total of US $150) as compensation for their participation.

## Results

### Design Phase I

#### Individual Interviews with Adolescents Diagnosed with Asthma

We conducted individual interviews with adolescents diagnosed with persistent asthma to identify what intrapersonal and external barriers to adherence to ICS are most salient to adolescents with asthma and to solicit their opinion about the types of intervention content that an mHealth intervention should deliver. We conducted the individual interviews with SRT [28] in mind; we asked about components of SRT if they were not mentioned or probed about how participants’ comments may be related to SRT. Multimedia Appendix 1 presents our individual interview guide questions. Table 1 presents the characteristics of the individual interview and advisory board participants. With regards to diversity, ≥50% of our sample was comprised of racial and ethnic minority groups.

**Table 1:** Youth, caregiver, and family demographic and medical characteristics of the interview and advisory board participants.

Characteristics		UF^a^ (n=13)	KU^b^ (n=10)
Youth age (years), mean (SD)		15.1 (1.04)	15.7 (0.95)
Caregiver age (years), mean (SD)		45.5 (10.90)	44.3 (8.22)
**Youth gender, n (%)**			
	Female	7 (54)	7 (70)
	Male	6 (46)	3 (30)
	Other	0 (0)	0 (0)
**Caregiver gender, n (%)**			
	Female	12 (92)	10 (100)
	Male	1 (8)	0 (0)
	Other		
**Youth race, n (%)**			
	Black/African American	3 (23)	0 (0)
	Caucasian	6 (46)	7 (70)
	Multiracial	4 (31)	3 (30)
**Youth ethnicity, n (%)**			
	Non-Hispanic/Latino	10 (77)	6 (60)
	Hispanic/Latino	3 (23)	4 (40)
Non-Hispanic Caucasian youth, n (%)		5 (38)	5 (50)
**Frequency of youth asthma attacks (past year), n (%)**			
	A few times a week	1 (8)	3 (30)
	A few times a month	6 (46)	4 (40)
	About once a month or less	6 (46)	3 (30)
**Youth asthma-related emergency department visits (past 4 weeks), n (%)**			
	0	12 (92)	9 (90)
	1	1 (8)	1 (10)
**Youth asthma-related emergency department visits (past year)**			
	0	6 (46)	8 (80)
	1	4 (31)	1 (10)
	2	1 (8)	1 (10)
	3	1 (8)	0 (0)
	5	1 (8)	0 (0)
**Youth asthma-related sick visits, mean (SD)**			
	Past 4 weeks	0.6 (0.9)	0.3 (0.7)
	Past year	2.6 (2.3)	2.7 (3.7)
**Youth school days missed due to asthma, mean (SD)**			
	Past 4 weeks	1.5 (4.1)	0.4 (1.7)
	Past year	9.9 (20.0)	1.7 (4.7)
**Frequency of youth quick relief medication use (past 4 weeks), n (%)**			
	Never	4 (31)	3 (30)
	0-2 days a week	5 (38)	4 (40)
	3-6 days a week	1 (8)	2 (20)
	Every day of the week	3 (23)	1 (10)
**Caregiver education, n (%)**			
	High school	5 (38)	4 (40)
	Some college	1 (8)	1 (10)
	College	3 (23)	4 (40)
	Graduate school	2 (15)	0 (0)
	Other	2 (15)	1 (10)
**Family income (US $), n (%)**			
	<12,000	0 (0)	2 (20)
	12,000-24,999	3 (23)	1 (10)
	25,000-49,999	4 (31)	3 (30)
	50,000-99,999	3 (23)	2 (20)
	≥100,000	1 (8)	1 (10)
	No response	2 (15)	1 (10)

^a^UF: University of Florida.

^b^KU: University of Kansas.

#### Individual Interview Themes

Table 2 includes the themes of intrapersonal and external barriers endorsed by the adolescents. Adolescents noted a range of intrapersonal barriers to ICS adherence including forgetting (19/20, 95%), difficulties with time management or having a busy schedule (16/20, 80%), and being too fatigued or tired (12/20, 60%). Notably, less than half our sample endorsed a range of additional intrapersonal barriers (eg, mood, stress, laziness). Regarding external barriers to adherence, 12 adolescents (12/20, 60%) reported changes to their routine (eg, being away from home) as barriers. Adolescents also frequently endorsed not having medication available (11/20, 55%) and interference from other activities (10/20, 50%).

Adolescents provided several suggestions about how an mHealth intervention could promote ICS adherence. All adolescents endorsed that an mHealth intervention should provide reminders to take ICS medications and suggested the frequency of notifications from an mHealth intervention should be each time a dose is scheduled (15/20, 75%) or to be flexible dependent upon need (13/20, 65%). They also generally agreed that an mHealth intervention should provide personalized notifications (13/20, 65%) and facilitate tracking of adherence over time (20/20, 100%). Finally, a number of adolescents endorsed that they were receptive to an mHealth intervention sending text messages (10/20, 50%) and including interactive videos (10/20, 50%) to deliver content.

**Table 2:** Adolescent-endorsed intrapersonal and external adherence barriers to inhaled corticosteroid adherence.

Interview themes		Participants who endorsed, n (%)		Number of times a barrier was mentioned		Description of barrier		Sample quotes	
**Intrapersonal barriers**									
	Forgetting		19 (95)		71		The teen describes forgetting to take their medicine.		“I definitely forget to take it at least 2-3 times a week.”
	Time management/busyness		16 (80)		35		The teen does not take medicine due to busyness or other activities (eg, going to school or work).		“Having to take medicine on a daily basis is a bit difficult for me because I have a busy schedule. I’ll be more focused on something and end up forgetting.”
	Sleep/fatigue		12 (60)		21		The teen does not take medicine due to abnormal sleep patterns, difficulty waking up in the morning, going to sleep late, or being fatigued.		“If I’m really tired or I had a long day or I got back from a soccer game, usually if it’s a late night, I’ll forget to take it because I’ll be so tired.”
	Mood		8 (40)		11		The teen’s mood (eg, depression, anxiety, sadness, frustration, anger) is a barrier that prevents them from taking medicine.		“If I’m kind of like in a ‘eh’ mood, I won’t take them, but if I’m happy, I’ll take them. Yeah, it definitely depends on my mood.”
	Not wanting to take meds		6 (30)		12		The teen mentions not wanting to, deciding not to, or not feeling like taking meds without giving a reason that would better fit in another category.		“Besides forgetfulness, sometimes I just don’t feel like taking it.”
	Stress		4 (20)		5		The teen describes stress as a barrier to taking medicine.		“I guess sometimes if I'm super tired or stressed, then I won't really focus on [taking meds].”
	Embarrassment		3 (15)		5		The teen describes embarrassment as a barrier to taking medicine (eg, being embarrassed to take medicine in front of friends).		“It’s kind of embarrassing to pull out an inhaler right before games… It’s just not being embarrassed to take it in front of other people I guess.”
	Laziness		3 (15)		3		The teen mentions laziness as a barrier to taking medicine.		“It’s just my own, I don’t know, laziness I guess that makes it harder to take my medication.”
	Not seeing meds as necessary		3 (15)		3		The teen does not see medicine as necessary (eg, they believe taking medicine does not help them, or they believe they do not need medicine).		“The controller one, it doesn't really do that much to you. It doesn't have an effect if you stop taking it or not. That's why it doesn't affect me that I didn't take it that much.”
	Feeling sick		2 (10)		3		The teen does not take medicine due to feeling sick (eg, nauseous, having a headache).		“I could be extremely nauseous one day and not take it for the reason that I would throw up if I did.”
	Misplacement		2 (10)		3		The teen does not take medicine because they or someone else lost their medicine (inhaler or spacer).		“One time [my dog] put my inhaler somewhere that I couldn't find it.”
	Feeling different		1 (5)		1		The teen does not take medicine because it reminds them that their asthma diagnosis makes them different from their peers.		“It’s depressing when you can’t run with the other kids in gym or you can’t take dance classes or you have to sit out of something or just do your own thing… You feel like you’re not normal.”
	Lack of organization		1 (5)		1		The teen mentions “not being organized” as a barrier to taking medicine.		“Not being organized [makes it harder to take meds], or like having so much stuff to do.”
**External barriers**									
	Changes in routine		12 (60)		24		The teen describes changes in their routine (eg, being away from home) as a barrier to taking medicine.		“Running late [makes it harder to take meds], and when it’s not the weekdays, because in the morning I just have my routine, but on the weekends it varies.”
	Running out of meds		11 (55)		11		The teen runs out of medicine because they forget to fill their prescription or they have insurance difficulties.		“I won’t check how many I have left, how many pills or QVAR puffs or something, I’ll run low, so I have to take one puff instead of two. Or I’ll run out before we can order it and get it back in.”
	Other things get in the way		10 (50)		21		The teen does not take medicine due to interference from various activities (eg, watching TV, caring for dog). These activities are often unspecified (eg, “doing other stuff”).		“Most of the time, it's hard for me to remember because I'll be thinking about other things, too, so it isn't really right there in the top of my head.”
	Friends, peers, or other people		7 (35)		10		The teen does not take medicine due to influence from other people, including peers (eg, hanging out with friends, feeling victimized by peers) and siblings.		“It could be times where I hang out with friends and stuff that could get in the way [of taking meds], because I'm going to forget about everything.”
	Changes in medication		1 (5)		1		The teen does not take medicine because they are in the process of changing their medicine, inhaler, or dosage.		“There was [a time] when I was in-between medicine on the one that I take every day, because they were changing the style of the inhaler and the doses, and I didn't take it for a little while.”
	Cost of medicine		1 (5)		1		The teen has difficulty obtaining medicine because it is too expensive.		“[My doctor] wants to put me on a new inhaler, but the insurance doesn't want to cover it, so my pharmacy has to talk to the doctor, and they're trying to figure that out, so I'm not able to use what the doctor wants me to right now.”

#### Overview of Intervention Content Creation

**Figure 2:**
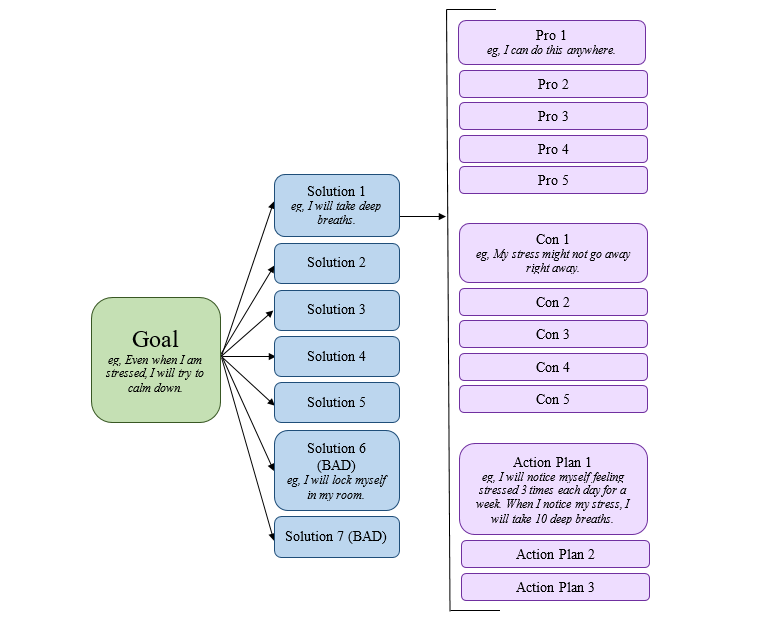
Example problem-solving workflow: stress.

We used information gathered from individual interviews in combination with our pilot data [29] and the broader asthma literature [30,31] to develop the following ReACT intervention content: animated videos, goal-setting messages, and problem-solving modules. We created custom animated videos to provide orientation to ReACT and national guidelines–based [4] asthma education [26]. Consistent with SRT [28], we created text message libraries to prompt self-monitoring, solicit intention formation for adherence goals, and provide feedback on adherence and goal progress. Messages were gain-framed [24] and delivered based on algorithms designed to consider the recent adherence patterns of the adolescent. Finally, consistent with data gathered from individual interviews and the extant asthma management literature [31], we created problem-solving modules for 7 domains: stress, family conflict, motivation, regimen, low mood, social support, and asthma knowledge. We created text messages to guide participants to problem-solve intrapersonal and external barriers to ICS adherence endorsed via ecological momentary assessment [36]. Specifically, each module included text messages to prompt participants to identify potential goals, solutions, pros and cons, and action plans for identified barriers. Please see Figure 2 for an example problem-solving module.

### Design Phase II

#### Crowdsourcing

See Table 3 for the participant demographics for those who participated in the crowdsourcing. Notably, our crowdsourcing sample was predominantly comprised of youth from racial and ethnic minority groups (219/257, 85%). Crowdsourcing participants rated 514 out of 555 (93%) problem-solving messages as not needing modification (ie., received <40% “no” votes from participants in the crowdsourcing phase). We accepted this intervention content as semifinal, although study staff made minor revisions to this content, when appropriate, based on themes that emerged during advisory board feedback (eg, modifications to messages). Of the 555 problem-solving messages, 33 (6%) received 40.0%-59.9% “no” votes, and we revised these messages in conjunction with the advisory board. We had 8 (8/555, 1%) problem-solving messages that received >60% “no” votes. We revised both the wording and content of these messages on a case-by-case basis (see Table 4 for crowdsourcing data). Frequently, crowdsourcing participants correctly identified “problematic” solutions within the problem-solving framework and gave it a no vote (eg, “I will get angry and blame everyone else for my stress”). If participants gave suggestions to modify these messages, study staff made modifications using a consensus process.

**Table 3:** Youth, caregiver, and family demographic characteristics of the crowdsourcing participants.

Characteristics		Youth (n=257)	Caregivers/family (n=257)
Age (years), mean (SD)		15.0 (1.34)	41.6 (7.64)
**Gender, n (%)**			
	Female	127 (50)	200 (78)
	Male	129 (50)	57 (22)
	Other	1 (<1)	0 (0)
**Race, n (%)**			
	Black/African American	113 (44)	113 (44)
	Caucasian	48 (19)	44 (17)
	Asian	39 (15)	42 (16)
	Multiracial	34 (13)	36 (14)^a^
	American Indian/Alaska Native	18 (7)	17 (7)
	Native Hawaiian/Pacific Islander	5 (2)	5 (2)
**Ethnicity, n (%)**			
	Non-Hispanic/Latino	207 (81)	207 (81)
	Hispanic/Latino	50 (19)	50 (19)
	Non-Hispanic Caucasian	38 (15)	38 (15)
**Caregiver highest degree, n (%)**			
	Less than high school	N/A	19 (7)
	High school/GED^b^	N/A	54 (21)
	Associate degree	N/A	55 (21)
	Bachelor’s degree	N/A	71 (28)
	Master’s degree	N/A	45 (17)
	Doctorate	N/A	5 (2)
	Professional (eg, MD^c^, JD^d^)	N/A	4 (2)
	Other	N/A	4 (2)
**Caregiver income (US $), n (%)**			
	<12,000	N/A	20 (8)
	12,000-24,999	N/A	19 (7)
	25,000-49,999	N/A	61 (24)
	50,000-99,999	N/A	79 (31)
	≥100,000	N/A	72 28)
	No response	N/A	6 (2)

^a^Caregivers were able to select more than one race; when they did, we classified them as multiracial.

^b^GED: General Educational Development.

^c^MD: Doctor of Medicine.

^d^JD: Doctor of Jurisprudence.

**Table 4:** ReACT crowdsourcing feedback about the messages.

Votes received	Family conflict, n (%)	Knowledge, n (%)	Motivation, n (%)	Routine, n (%)	Social support, n (%)	Stress, n (%)	Low mood, n (%)	Total, n (%)
<40% “no” votes	76 (95)	68 (87)	69 (91)	75 (91)	76 (93)	69 (91)	81 (100)	514 (93)
40%-60% “no” votes	4 (5)	6 (8)	7 (9)	7 (9)	4 (5)	5 (7)	0 (0)	33 (6)
>60% “no” votes	0 (0)	4 (5)	0 (0)	0 (0)	2 (2)	2 (3)	0 (0)	8 (1)

#### Advisory Board Feedback

During the first advisory board meeting, we introduced core ReACT functionality, provided a study timeline and overview, and described the members’ role in helping to refine the intervention content and functionality of ReACT. We reviewed themes that emerged from Design Phase I and findings from the crowdsourcing data gathered as part of Design Phase II. For homework between the first 2 meetings, adolescents were asked to review and reword select intervention content that received 40%-60% “no” votes from crowdsourcing participants. During the second meeting, the group discussed the adolescents’ homework responses and worked together to further refine the intervention content. Specifically, advisory board members provided suggestions on how to modify message wording (eg, using more colloquial language) and generated additional content (eg, additional solutions) for inclusion in the problem-solving modules. We dedicated the final advisory board meeting to demonstrating the features of the core ReACT intervention elements such as intention formation, feedback, problem solving, barrier identification, and motivational assessment. During this meeting, advisory board members interacted with our adherence sensor and watched the ReACT orientation and asthma education videos, and we provided them full examples via an interactive computer presentation of the ReACT interface and intervention components. The advisory board provided positive feedback regarding the look and feel of the adherence sensor. The advisory board suggested several improvements to the videos, including reducing the length of the asthma education and increasing the focus on the role of adherence to ICS in asthma management. The advisory board provided predominantly favorable feedback regarding the timing and frequency of participant interactions with ReACT. They provided suggestions on how to improve intervention messaging (eg, temper enthusiasm in messages) and sequencing.

## Discussion

### Principal Findings

Our goal with ReACT was to involve stakeholders throughout the creation and refinement of intervention content. In this way, we sought to fill a gap we identified in prior reviews of the digital intervention literature [37,38]. Consistent with best practices in mHealth intervention development [26], we used both qualitative approaches with small samples (ie., advisory boards and individual interviews) and large-sample crowdsourced feedback to develop our intervention content. The feedback we received from participants largely supported our approach. Consistent with SRT and the extant asthma literature [31,39], adolescents frequently endorsed intrapersonal and external barriers to adherence to ICS and provided generally positive impressions regarding the use of mHealth as an intervention format. We view that the variability in the types of barriers endorsed during individual interviews (eg, mood, stress, forgetfulness) as evidence that an adaptive and individually tailored mHealth system, like ReACT, may be especially beneficial for adolescents with asthma. Of note, adolescent feedback regarding barriers did not address the frequency of barriers (eg, forgetting to take medicine, feeling stressed). More research is needed to determine the barriers’ frequency and impact on objective adherence behaviors.

Adolescents suggested improvements to ReACT intervention content in several instances. Similar to other intervention development studies, the main lesson learned from this process was the importance of engaging target users to increase the likelihood that our intervention content is communicated in a way that is relevant to adolescents with asthma. Refinements in intervention content came in 2 forms. First, participants helped with message clarity and ensured that our team, comprised of adult academicians, was effectively communicating behavior change concepts in language adolescents could understand [35]. Second, adolescents helped with the tone of the messages. There was a tendency among our team to generate excessively enthusiastic messages in an attempt to ensure that participants have a pleasant experience in the intervention. Our development phase revealed that some adolescents perceived this style as disingenuous. As a result, we removed exclamation points and overly enthusiastic phrases to make our messages more matter-of-fact, while still supportive.

Another important lesson learned from this development phase was that problem solving can be challenging to convey in text messages. A critical element of learning to effectively solve a problem is to briefly entertain goals and solutions that might not be productive over the long-term and may not ultimately be selected for implementation. However, our crowdsourcing participants seemed to vary in the degree to which they understood this concept. All of the messages that received a high number of “no” votes were “problematic” goals or solutions that we intended to help illustrate this component of the problem-solving process. Free-form responses made it clear that participants in the crowdsourcing were correctly identifying the message as “problematic” but appeared to misunderstand the intentional decision to include nonproductive goals and solutions as part of the problem-solving process. This confusion may have limited the frequency and depth of feedback we received during the crowdsourcing phase.

### Limitations

We acknowledge several limitations with the current study. First, we recruited participants for individual interviews and the advisory board via convenience sampling methods. Our advisory board was comprised of a small, racially and ethnically diverse sample (n=4) of adolescents with persistent asthma from one site. Therefore, although advisory board feedback was helpful in refining messages, it is possible that participants’ feedback on the intervention content is not generalizable to the larger population of adolescents with asthma. These limitations should be considered in light of involving target users in the development of ReACT from 2 study sites and gathering nationally representative feedback on ReACT intervention content from a crowdsourcing process in which 85% of youth were from racial and ethnic minority groups. Given well-documented health disparities among youth from racial/ethnic-minority youth [40], it is critical to engage a diverse sample of target end users in the design of mHealth interventions like ReACT. We posit that crowdsourcing may be a viable method to increase diversity in future mHealth intervention development studies. We acknowledge that we did not collect data on youth ICS use patterns during the crowdsourcing phase. Thus, we are unable to examine potential associations between user preferences for intervention content and self-reported ICS use. It is also noteworthy that caregiver stakeholders in the individual interview and advisory board portions were primarily female. While this likely reflects typical care patterns, we may be missing valuable perspectives from male caregivers.

### Future Research

Our immediate next steps are to conduct a pilot acceptability, usability, and preliminary efficacy study with target ReACT users [23]. Specifically, our pretest-posttest design will include a sample of 20 adolescents with persistent asthma. They will complete a 4-week baseline ICS monitoring-only period followed by a 4-week ReACT intervention period. We will gather data on enrollment rates and usage statistics and monitor technical difficulties to evaluate feasibility of ReACT. Acceptability and usability will be determined via questionnaires and a semistructured interview that asks adolescents to discuss the perceived usefulness of ReACT, how effective the intervention was in changing asthma self-management, and what changes we should make to ReACT in advance of further testing. Finally, we will evaluate preliminary efficacy by exploring changes in our hypothesized mediational variables (eg, self-efficacy) and adherence to ICS

## Appendix

Multimedia Appendix 1 Individual Interview Guide Questions.

## Abbreviations

GED: General Educational Development.
ICS: inhaled corticosteroids.
JD: Doctor of Jurisprudence.
MD: Doctor of Medicine.
mHealth: mobile health.
ReACT: Responsive Asthma Care for Teens.
SRT: self-regulation theory.
