# Supplementary material for: Adaptive Mobile Health Intervention for Adolescents with Asthma: Iterative User-Centered Development
Source: JMIR Mhealth Uhealth. 2020 May 6;8(5):e18400. doi: 10.2196/18400 (PMC7240449; doi:10.2196/18400)
Supplement: Multimedia Appendix 1 [file mhealth_v8i5e18400_app1.docx]

**ReACT Individual Interview Guide**

**Section 1. Background**

1. I’d like to start by asking how asthma fits into your day. How has having daily asthma medication prescribed for you changed the way you and your family go about your day-to-day lives? [*Probe about challenges, sharing of treatment responsibilities (i.e., who is responsible for taking meds?)*]
   1. How has it caused challenges to your daily routine? (e.g., needing to do an additional thing each day)
   2. How has taking care of your asthma impacted what you and your family talk about?
   3. Who is responsible for making sure you are taking the medicine every day?

**Section 2. Identification of Barriers**

1. I want to talk about taking care of your asthma. What has your doctor has asked you to do to take care of your asthma? [*If participant is unclear what you’re asking, provide examples of things health care team may say, like specific times of day to take medicines, what to do when you’re away from home, etc. Focus on meds.*]
   1. In the previous question, you said ____. What else has the doctor told you to do?
   2. What suggestions did the doctor give you about taking your meds when you are away from home?
   3. [*If not mentioned*] Did the doctor tell you to take the medicine during a specific time of the day?
2. Some kids have a difficult time doing exactly what their doctors ask all of the time. What makes taking your daily asthma medication hard at times? [*Probe for difficulties with ICS (e.g., knowledge, forgetfulness, concerns about taking medicine all the time)*]
   1. Some people might be concerned about taking medicine all the time. How about you?
   2. Some people might only take their medicine when they have symptoms. Does this happen to you? [*Probe for knowledge regarding ICS*]
   3. [*If not mentioned*] When you are busy with other activities, do you forget about taking the medicine at times?
3. What people or things in your life have made it easier for you to take your daily asthma medication? [*Probe for social and family supports*] How about people or things that have made it harder to take your medicine?
   1. [*If not mentioned*] What does your family do to help you take your asthma medicine?
   2. What about other people in your life, like friends?
4. What things get in the way of taking your daily asthma medication? [*Probe for mood, stress, forgetfulness, access to care, availability of medications, resources, caregiver involvement*] What things remind you or help you to do what you need to in order to take care of your asthma?
   1. How does being in certain moods (e.g., angry or sad) impact whether you take your medicine? *(mood)*
   2. What about stress? When you are going through a lot of things in your life and you are stressed, how does this impact taking your medicine? *(stress)*
   3. Tell me about when you forget about taking your medication. *(forgetfulness)*
   4. What about during the occasions where you are away from home say for a sports event, friend’s house, or field trip? How have times like this impacted taking your medicine? Could you give me an example? *(availability of meds)*
   5. Sometimes we run out of the medicine and can’t get a new inhaler right away. When has that happened to you? *(access to care)*
   6. In general, how are your parents or caregivers involved with your asthma medicine? *(caregiver involvement)*
5. If participant is not motivated to take medications: It sounds like you are not sure you want to be taking asthma medicine every day. Are there things that would make you feel differently about that or more motivated in taking it?

If participant is motivated: It sounds like you are or you’d like to be taking your medicine more regularly – what would you do when things get in the way? [*Probe for problem solving strategies*]

1. What other strategies can you think of to overcome these challenges?

Have you ever set goals for taking your medication? [*If confused, probe: “You know how people often make New Year’s resolutions?”*]

If Yes: How do you go about it?

1. How would you know if you are meeting the goals?

If No: If you decided to do that, how would you go about it?

1. How would you know if you are meeting the goals?

**Section 3. ReACT Content**

Okay, now we’re going to switch gears and talk about the phone app I mentioned earlier. We are very interested in hearing your thoughts on how an app could help keep teens on track with taking their asthma medicines.

1. If you were going to use an app for asthma to help teens take their asthma medicines, what would you want it to do? [*Probe for how an app could help meet a teen’s goals for asthma care*]
   1. We wanted to create an app to help teens to better take care of their asthma. But right now we don’t know what’s the most important things to include. You mentioned that sometimes [barrier] gets in the way of taking your medication and that an app could [solution] to help. These are the types of things we are looking to include in the app. What other things do you want this app to do in order to help you with your asthma?
2. In your opinion, how might an app help someone with asthma with [*List barriers mentioned in Section 2; don’t need all probes for every participant*]?
   1. How might an app help a teen take their asthma medicine if they are in a bad mood? *(mood)*
   2. What about when they are feeling stressed? If this happens, what do you think the app can do to help them better manage their asthma? *(stress)*
   3. If someone is not taking their asthma medicine every day because they often forget about it, how would you think the app could help them remember to take it? *(forgetfulness)*
   4. Would it be helpful to have a parent or caregiver involved in an app like this? Why or why not? *(caregiver involvement)*

[*Combine the following prompts if needed:*]

- 1. What if the medicine is not immediately available to you because you are out on a field trip? How could the app help you prevent things like this from happening again? *(availability of meds)*
  2. During the times when you run out of your medicines and can’t get the new ones right away, what would you want the app to do to solve this issue in the future? *(access to care)*
  3. What if you don’t have a ride to the doctor’s office or your car’s not available to you when you need to go to the doctor’s office? What things do you want the app to do to help you avoid these problems? *(resources)*

1. How would you want to get information from an app (e.g., text messages, videos)? Why?
   1. Do you want to get the information in text messages, video representations, or pictures? Which do you prefer? Why?
2. How often would you want an app to send you notifications or information? Would that change depending on the day or what is going on in your life at the moment?

**Section 4. Using an App**

1. What would get in the way of using an app for asthma? [*Probe for practical barriers (e.g., school, access to phone) and personal barriers (e.g., general low interest, annoyance from app reminders)*]
   1. Sometimes you may have limited access to your phone. For example some schools do not allow students to use their phones while in schools. Do you think that would get in the way of using the app? *(school)*
   2. Are there any other occasions where you would not be able to use the app because you don’t have access to your phone? *(access to phone)*
   3. Some apps send you notifications all the time. Would you be annoyed if our app does this? *(annoyance)*
   4. In general, if you are just not interested in using this app at all, would you stop using it? (low interest)
2. How could we get teens interested in participating in a project to use an app for asthma?
   1. What do you think about using flyers?
   2. How about using social media to spread the news about the study?
